# Gait characteristics under different walking conditions: Association with the presence of cognitive impairment in community-dwelling older people

**DOI:** 10.1371/journal.pone.0178566

**Published:** 2017-06-01

**Authors:** Anne-Marie De Cock, Erik Fransen, Stany Perkisas, Veronique Verhoeven, Olivier Beauchet, Roy Remmen, Maurits Vandewoude

**Affiliations:** 1 Department of Geriatric Medicine, General Hospital St Maarten, Mechelen, Belgium; 2 Department of Geriatrics and Department of Primary and Interdisciplinary Care (ELIZA), University of Antwerp, Antwerp, Belgium; 3 StatUa Center for Statistics, University of Antwerp, Antwerp, Belgium; 4 Department of Medicine, Division of Geriatric Medicine, Centre of excellence on aging and chronic disease (CEViMaC), McGill University, Montreal, Canada; Northwestern University, UNITED STATES

## Abstract

**Background:**

Gait characteristics measured at usual pace may allow profiling in patients with cognitive problems. The influence of age, gender, leg length, modified speed or dual tasking is unclear.

**Methods:**

Cross-sectional analysis was performed on a data registry containing demographic, physical and spatial-temporal gait parameters recorded in five walking conditions with a GAITRite® electronic carpet in community-dwelling older persons with memory complaints. Four cognitive stages were studied: cognitively healthy individuals, mild cognitive impaired patients, mild dementia patients and advanced dementia patients.

**Results:**

The association between spatial-temporal gait characteristics and cognitive stages was the most prominent: in the entire study population using gait speed, steps per meter (translation for mean step length), swing time variability, normalised gait speed (corrected for leg length) and normalised steps per meter at all five walking conditions; in the 50-to-70 years old participants applying step width at fast pace and steps per meter at usual pace; in the 70-to-80 years old persons using gait speed and normalised gait speed at usual pace, fast pace, animal walk and counting walk or steps per meter and normalised steps per meter at all five walking conditions; in over-80 years old participants using gait speed, normalised gait speed, steps per meter and normalised steps per meter at fast pace and animal dual-task walking. Multivariable logistic regression analysis adjusted for gender predicted in two compiled models the presence of dementia or cognitive impairment with acceptable accuracy in persons with memory complaints.

**Conclusion:**

Gait parameters in multiple walking conditions adjusted for age, gender and leg length showed a significant association with cognitive impairment. This study suggested that multifactorial gait analysis could be more informative than using gait analysis with only one test or one variable. Using this type of gait analysis in clinical practice could facilitate screening for cognitive impairment.

## Introduction

Gait is a complex action composed of a cyclic movement, changing support and balance from one foot to the other. It is influenced by muscular strength and performance, peripheral neuronal activation and control, but also by central neural commanding control [1,2]. Reduced motion may therefore be indicative of neuronal degeneration and/or decline in physical performance.

Gait speed is used as a screening parameter for multiple geriatric syndromes [3] like sarcopenia or frailty [4]. Currently, however, according to the definitions of these geriatric syndromes, gait speed is the only gait parameter used [5]. Gait speed is proposed as a screening criterion for cognitive decline in Motor Cognitive Risk (MCR) Syndrome and mild cognitive impairment (MCI) [6]. Recent work from the Motor Cognitive Risk (MCR) Consortium revealed that gait speed seems to have early detection abilities for cognitive impairment[7]. Participants had no cognitive impairment, but only memory complaints. The MCR subjects were detectible due to their lower gait speed at usual pace. However, evidence of the added value of these findings in clinical setting is still lacking. Previous researchers suggested that this could be due to the low sensitivity en specificity when gait speed is used as a single parameter[8]. Further exploration of the gait cycle may result in the detection of more useful single or clusters of gait characteristics and ensue a better understanding how gait behaves phenotypically in different age groups and in geriatric syndromes. Using gait characteristics that are related to specific neurologic regions in the brain affected by dementia may result in higher sensitivity and/or specificity to detect the cognitive declining subjects. Studying spatial-temporal gait characteristics has become more feasible due to the simplicity and the low cost of computerised gait examination [9].

The Gait, Cognition and Decline (GOOD) initiative, went one step further by studying gait characteristics other than gait speed alone in the diagnosis of cognitive decline [10]. This cross-sectional multicentre project, using a similar computerised GAITRite® set-up and device, listed the following gait variables usable for profiling. When severity of dementia increased, gait slowed down and characteristics changed. Stride velocity was strongly related to dementia severity and high mean and co-variability of step length were related to moderate dementia. High variability in stride time was related to mild cognitive impairment (MCI). Based on this study, these spatial-temporal gait characteristics were deemed meaningful for screening purposes.

Limitations of the GOOD initiative were the lack of uniformity in test set-up and in patient selection. The multicultural nature of the patients may have influenced gait patterns, and the groups were not age stratified. The test speed was also assumed to be at usual pace, although the speed mode was not clearly reported. The body height or leg length, influencing the speed and step length of an individual was not taken into account. Frimenko et al suggested that size plays a role in gait speed differences between genders, particularly the larger step length in men and higher cadence in women [11]. Further, using dynamic parameters for the detection of cognitive impairment was never considered. These limitations make the results less suitable for generalisation.

In the current study, we aimed to clarify the impact of age and leg length on the association of spatial-temporal gait characteristics with different levels of cognitive impairment. We explored if the use of different walking conditions had additional value in screening and predicting the presence of cognitive impairment. We hypothesised that 1) stratification for age would reveal other relationships between gait and cognition, as age influences gait performance and stability, 2) normalised gait speed and step length would eliminate the gender effect and yet preserve the differentiation ability of these parameters and 3) a standardised multiple-walking-condition-test configuration would create more differentiation variables and predictive abilities for the severity stages of cognitive impairment.

## Materials and methods

### Participant selection criteria, physical assessment and questionnaires

All people older than age 50 years attending the Memory Clinic in Mechelen, Belgium, were eligible for inclusion. Consecutive patients were entered prospectively in a database from April 2010 until November 2015.

All patients were assessed according to standard dementia screening and diagnosis protocols using a full cognitive test battery and physical evaluation. Participants were excluded when dementia was severe in accordance with a Mini Mental State Evaluation (MMSE, [12]) of less than 11, when the participant was unable to walk 10 meters without a walking aid or when a language barrier prevented further testing. The participants had to be still living at home.

The demographic status, medical history, social status, care support and Rockwood’s frailty index [13] were registered. These data included: age, gender, ethnic background, education level, disability using Activity of Daily Living (ADL or Katz) scoring [14] and instrumental ADL (iADL) function evaluated according to Lawton/Brody [15], number of medications and Timed Get Up and Go test (tGUG)[16,17]. Fall risk was assessed using three parameters: by asking the question: “did you fall during the last 12 months”, the Timed Chair Stand test (TCST) as part of the Short Physical Performance Battery [18] and the Functional Reach test (FR)[19]. The tGUG test, TCST and FR test are validated tests used in geriatric examination and considered good practice in the geriatric assessment for falls. These tests should be performed in stable clinical conditions [20–23].

From participants’ medical records, we obtained information on the presence of depression, cardiac ischaemia, heart failure, hypertension, cerebrovascular ischaemia, diabetes, chronic obstructive lung disease and gait disorders (Parkinson’s disease, parkinsonism or arthritis).

The cognitive screening included the MMSE, ‘Addenbrook’s cognitive evaluation- revised’ translated in Dutch (ACE-R) The combination of these two screening methods has good accuracy, clinical utility and is currently accepted as good practice in screening for cognitive decline in clinical settings. The combination also provides information on the cognitive domains and differentiation whether or not cognitive impairment is present [24–26]. The WAIS-IV neuropsychological battery to establish the dementia diagnosis according to the National Institute on Aging and the Alzheimer's Association workgroup revised criteria for Alzheimer's disease, dementia types and pre-dementia stages [27–29]. Using these criteria, we defined six groups: Amnestic mild cognitive impairment, non-amnestic mild cognitive impairment, Alzheimer type dementia, mixed/vascular dementia, Lewy body Disease and Fronto-temporal lobe dementia.

The psychological status and behaviour of the test participants was registered in two ways. Firstly, by questioning the participant about current complaints or history of depression, and secondly by administering a Neuropsychiatric Inventory (NPI) questionnaire [30].

Blood was taken in the morning after overnight fasting to measure haemoglobin, calcium, cyanocobalamin (vitamin B12), folic acid, 25-hydroxycholecalciferol (vitamin D), albumin levels, creatinin clearance, cortisol and parathyroid hormone levels. Brain imaging using Multi-slice Computer Tomography was coded for the presence of white matter lesions (yes or no) and the region of cortical atrophy (none, frontal, parietal/temporal, global) by one geriatrician and one radiologist. When clinically indicated, a fludeoxyglucose (^18^F)—positron emission tomography (FDG-PET) scan was performed to refine the diagnosis.

Dementia level and dementia type was defined for each patient in consensus meeting with the clinical dementia expert group (geriatrician, neurologist, radiologist, occupational therapist and neuropsychologist) at the Memory Diagnosis Centre according to DSM-IV criteria and using two severity scales, the Clinical Dementia Rating scale (CDR) (CDR code: 0 = Normal; 0.5 = Very Mild Dementia; 1 = Mild Dementia; 2 = Moderate Dementia; 3 = Severe Dementia) [31] and the Global Deterioration Scale (GDS) (Stage 1: No cognitive decline; Stage 2: Very mild cognitive decline; Stage 3: Mild cognitive decline; Stage 4: Moderate cognitive decline; Stage 5: Moderately severe cognitive decline; Stage 6: Severe cognitive decline; Stage 7: Very severe cognitive decline) [32].

The current research results are part of a larger project, investigating the significance and complexities of gait analysis in cognitive screening and diagnosis and in the present study the CDR code was used as stratification method.

### Technical investigations

Gait analysis was performed on a 6.1 meter computerised walkway with embedded pressure sensors (GAITRite® platinum, CIR systems, Havertown, PA, USA) permanently installed in a test room equipped in agreement with the GAITRite® users group criteria in a quiet, indirectly lit room and with participants wearing their daily footwear [9]. The GAITRite® system is widely used in clinical and research settings and has excellent validity and reliability [33,34]. Participant leg length was measured from the top of the greater trochanter to the ground on both legs. Leg length normalisation was used to correct parameters influenced by size and presumably by gender [11]. Participants were instructed to walk along the walkway, starting two meters before and stopping two meters after the walkway, marked as starting point and end point respectively, in five different walking conditions. We named this ‘the 5-Walk test method’. A different instruction was given before each walk. First walk: ‘Walk from the starting point to the end point at your usual pace like you would walk in the street’. This walk was marked as ‘usual pace’ (UP). Second walk: ‘Walk from the starting point to the end point as fast as you can without running’. This second walk was marked as ‘fast pace’ (FP). Third walk: ‘Walk from the starting point to the end point as slow as you can without standing still’. This third walk was marked as ‘slow pace’ (SP). Fourth walk was a dual task: ‘Walk from the starting point to the end point at your usual speed and count down aloud starting from fifty in steps of two’. This fourth was marked as ‘counting walk’ (CW). Fifth walk was also a dual task: ‘Walk from the starting point to the end point at your usual speed and name aloud all animals you know’. This fifth walk was marked as ‘animal walk’ (AW).

The GAITRite® computer software 4.8.3 Designer automatically calculated the gait speed in centimeters (cm) per second (s), cadence in steps per minute, mean step width in cm, step width variability in percentage, swing time (and cycle time variability in percentage. The gait speed of every walk was normalised according to leg length in meter per second (m/s). The number of steps per meter, a translatable measure of the mean stride length of every walk, was calculated dividing cadence by gait speed or normalised gait speed (steps per meter or normalised steps per meter). Dual-task-cost (DTC) of every dual task gait variable was calculated with reference to the usual pace gait parameters calculating the percentage difference between the two modes (‘Usual pace’ minus dual task walk divided by the ‘usual pace’ parameter).

### Ethics committee

Every participant signed an informed consent (IC). This IC procedure is part of the quality procedure maintained in the memory clinic to insure that patients and relatives are aware of the diagnostic pathway they will follow. For participants with cognitive impairment the information is given to the patient and the legal guardian. The signed document is also obtained from the patient and the legal guardian. The ethics committee of Emmaus—St Maarten General Hospital Mechelen approved the study design in 2012 as a retrospective study on patient data using the standard procedure in dementia diagnosis customised with gait analysis in the GAITRite® system (Emmaus EC 1218).

### Statistical analysis

Participants were grouped into four cohorts based upon the CDR levels. Cognitively healthy individuals (CHI = CDR 0), Mild cognitive impaired (MCI = CDR 0.5) people were assigned to the first and second group, mild dementia patients (CDR 1) were assigned to group three and the fourth group renamed 'advanced dementia' consisted of moderate to severe dementia patients (CDR 2 and 3). Cognitively healthy individuals were considered as cognitively normal when clinical criteria for cognitive dysfunction were not met.

The association between CDR level and the clinical characteristics were tested using a one-way ANOVA for continuous variables and using Chi-square test for categorical variables. The analysis was performed on the entire study population as well as on an age-matched group comprising 50 to 70 year old participants, 70 to 80 year old participants and a group consisting of only over-80-year old participants. Continuous variables are expressed as mean values ± standard deviation. The association between the cognitive indicators (independent variable) and the gait parameters (dependent variables) for all five walking conditions was computed using one–way ANOVA and chi-squared test. P-values were considered significant when p ≤ 0.05.

In the multivariable analysis, a two-way ANOVA model was fitted with the gait parameters as the dependent variable (for the five walking conditions separately), and as the independent variables the CDR score and gender. The p-values for the association between the outcome and the CDR score are reported for the entire group and split by age category. In addition, we tested for the interaction between gender and CDR score. P-values were considered significant when p ≤ 0.05.

For the statistical analysis on gait variability parameters (step width variability, cycle time variability, and swing time variability) and dual-task-cost analysis, we used data of participants performing a UP with normalised gait speed higher than 0.8 m/s. This is justified because slow gait speed is known to create unsteady variability parameters and has no added value on the discriminative power of these gait parameters [6,35].

To model how the gait parameters relate to the diagnostic groups, logistic regression models were fitted from normal individuals and people with varying degrees of dementia, using spatial-temporal gait parameters as independent variables. The five most discriminating gait parameters from previous calculations were selected (gait speed, normalised gait speed, steps/meter, normalised gait speed, swing time variability) at the five walking conditions (UP, FP, SP, CW and AW). Dementia diagnosis (yes or no) was entered as dependent variable in the first model. In a second model CDR code was entered as dependent variable using a differentiation between CDR 0 and CDR > 0, discriminating the CHI from cognitively affected groups. Univariate logistic regression analysis was calculated for each gait parameter separately for each of the five conditions. ROC curves and the area under the curve (AUC) were calculated. Multivariate logistic regression analyses models were fitted using a stepwise backward approach, starting form a model with all gait parameters and conditions and adding age as a covariate. Gender was added to the second model as a covariate.

Proportional Odds ordinal regression calculating the predicted probability to be included in a specific CDR group was also performed in the five parameters at five different walking conditions.

The data were analysed using JMP Pro 13.0 (SAS Institute) and the statistical package R version 3.1.2. (Software R by Core Team, 2014).

## Results

Over a period of five years, 535 participants (61% of 877 eligible people) were included for the analyses. Reasons for the exclusion of 342 people were: refusing to participate in several standard tests, not consenting to the standard procedure, under age 50 years old, severely demented, unable to walk without help, living in a nursing home, not able to perform the tests due to physical frailty.

The population characteristics of the four dementia-severity groups (CHI, MCI, Mild, Moderate) and their age-split and -matched subgroups are shown in the table in S1 Table.

In the entire study population, dementia severity increased with age (*F* (3,533) = 16.08, p <0.001). Over two-thirds of the patients aged 80 and over presented at the Memory Diagnosis Centre with mild to advanced dementia. Male participants were less represented in the dementia groups as severity rose (*X*^*2*^ *(3*,*N = 536) = 7*.*89*, *p = 0*.*05)*. This was due to the ageing of the groups. Years of education tended to be higher in the pre-dementia stages and decreased as dementia was more pronounced (*F* (3,533) = 16.07, p <0.001). Behavioural problems tended to be more severe as dementia severity increased (*F* (3,433) = 5.16, p = 0.002). Comorbidities such as depression, hypertension and COLD were equally present in all cognitive stages. Diabetes was more present in the pre-dementia stage (*X*^*2*^ (3,N = 536) = 15.52, p = 0.001). These differences were consistent across age-split and -matched subgroups.

Fall risk scores (TGUG, TCST and FR) indicated a lower risk for falling in the pre-dementia stages, although the effect was partially confounded with age (*F* (3,67) = 3.94, p = 0.01, *F* (3,196) = 2.49, p = 0.06, *F* (3,24) = 1.28, p > 0.05 respectively). Only a relation trend between enquired numbers of falls incidents and cognitive stage (*X*^2^ (3, N = 536) = 7.95, p = 0.05 in the total study population, *X*^2^ (3, N = 278) = 7.19, p = 0.07 in over 80 year old participants) was seen. This was probably due to inadequate reporting of events in advanced demented subjects disturbing these results.

Disability and frailty worsened in parallel with the dementia stage (*F (3*,*262) = 12*.*67*, *p* < 0.001). Rockwood’s Frailty index did not exceed the fifth level in most pre-dementia cases in all age groups. The Lawton score (complex daily living tasks) decreased in parallel with the dementia progression (*F (3*,*208) = 9*.*22*, *p* < 0.001). The Katz score (basic tasks of daily living) differed significantly between the four groups (*F (3*,*252) = 4*.*66*, *p =* 0.003). Differences in scores were minimal due to the community-dwelling characteristics of the cohort. These associations remained consistent across age-split and -matched subgroups.

The specific research questions were analysed as described below.

### Spatial-temporal gait characteristics in five different walking conditions at four different cognitive impairment stages, age-stratified and the effect of normalisation for leg length

**Fig 1:**
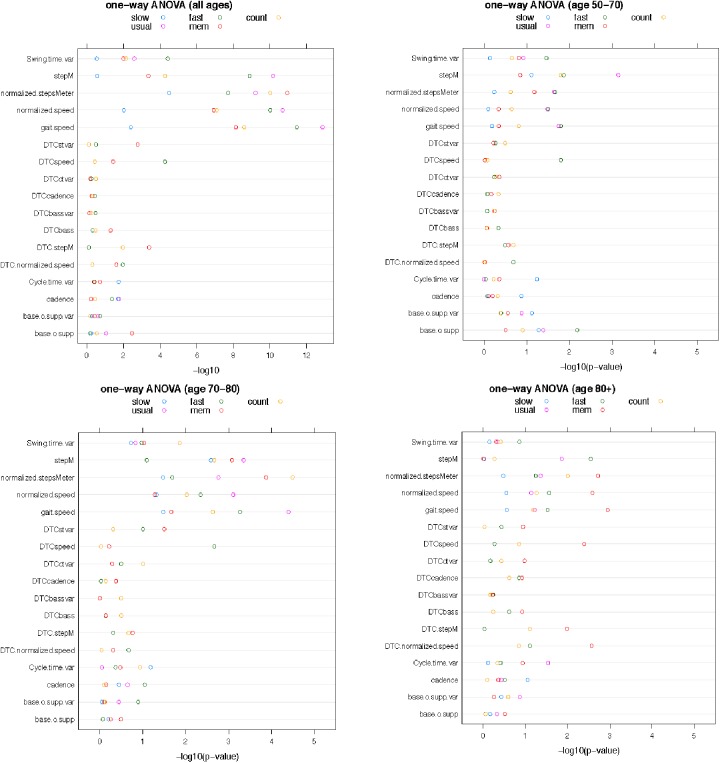
Dotplot: Association between dementia stages (CDR code) and gait variables (one-way ANOVA). Dotplot_all: Association between CDR score and gait (all ages combined). The dotplot shows the negative logarithm (10-based) of the p-values for the one-way ANOVA between gait parameter and CDR score. Strong associations with a small p-value correspond to large values of the–log(p). Each line in the plot corresponds to one gait parameter. On each line, five dots are shown for the 5 walking conditions. Dotplot_5070: Association between CDR score and gait (age 50 to 70). Dotplot_7080: Association between CDR score and gait (age 70–80). Dotplot_80plus: Association between CDR score and gait (above age 80). CDR: Clinical Dementia rating. DTC: dual task cost.

The one-way ANOVA showed associations between gait variables and cognitive stages (Fig 1 and S2 Table).

Gait speed as well as normalised gait speed at usual pace (UP) was significantly different between cognitively healthy, pre-demented people and those in dementia stages in the entire group (*F* (3,521) = 22.3, p <0.001); *F* (3,514) = 18.5, p <0.001, respectively). The difference remained in other walking conditions (fast pace (FP): *F* (3,510) = 19.9, p<0.001; *F* (3,504) = 17.4, p <0.001; animal walk (AW): *F* (3,483) = 14.2, p<0.001; *F* (3,477) = 12.1, p <0.001; counting walk (CW): *F* (3,473) = 15.0, p<0.001; *F* (3,465) = 12.4, p <0.001), except in slow walk mode (*F* (3,503) = 4.5, p = 0.004; *F* (3,499) = 3.85, p = 0.01). In age-stratified groups, gait speed and normalised gait speed in single tasking walk only UP and FP and not SP were significantly different between the CDR groups (UP: 50-to-70y gait speed *F* (3,68) = 3.62, p = 0.02, normalised gait speed F (3,68) = 3.10, p = 0.03; 70 to 80y gait speed *F* (3,222) = 8.07, p < 0.001, normalised gait speed *F* (3,218) = 5.81, p<0.001; Over 80y gait speed *F* (3,229) = 2.51, p > 0.05; normalised gait speed *F* (3,226) = 2.37, p > 0.05; FP: 50-to-70y gait speed *F* (3,68) = 3.71, p = 0.02, normalised gait speed F(3,68) = 3.14, p = 0.03; 70 to 80y gait speed *F* (3,220) = 6.09, p < 0.001, normalised gait speed *F* (3,217) = 4.49, p = 0.004; Over 80y gait speed *F* (3,220) = 3.06, p = 0.03; normalised gait speed *F* (3,226) = 3.11, p = 0.03), while dual tasking walks (AW and CW) were significant in all age groups, except for the 50-to-70-year olds (AW: 50-to-70y gait speed *F* (3,66) = 0.89, p > 0.05, normalised gait speed F(3,66) = 0.88, p > 0.05; 70 to 80y gait speed *F* (3,215) = 3.30, p = 0.02, normalised gait speed *F* (3,212) = 2.62, p = 0.05; Over 80y gait speed *F* (3,200) = 5.56, p = 0.001; normalised gait speed *F* (3,197) = 4.93, p = 0.003; CW: 50-to-70y gait speed F(3,62) = 1.84, p > 0.05, normalised gait speed *F* (3,61) = 1.49, p > 0.05; 70 to 80y gait speed *F* (3,207) = 4.99, p = 0.002, normalised gait speed *F* (3,204) = 3.93, p = 0.01; Over 80y gait speed *F* (3,202) = 2.46, p > 0.05; normalised gait speed *F* (3,198) = 2.59, p = 0.05).

Mean step length, expressed in our study as number of steps per meter, also differed across the dementia stages. Steps per meter, as well as normalised steps per meter, in UP differed among cognitively healthy, pre-demented people and those in dementia stages in the entire group (UP: Steps/meter *F* (3,378) = 18.0, p <0.001); normalised steps/meter *F* (3,511) = 16.0, p <0.001, respectively). The difference remained in the other walking conditions (FP: Steps/meter *F* (3,468) = 15.5, p<0.001; normalised steps/meter *F* (3,502) = 13.4, p <0.001; SP: Steps/meter (*F* (3,101) = 1.32, p>0.05; normalised steps/meter *F* (3,495) = 8.03, p <0.001); AW: Steps/meter *F* (3,149) = 6.38, p<0.001; normalised steps/meter *F* (3,476) = 19.1, p <0.001; CW: Steps/meter *F* (3,163) = 8.00, p<0.001; normalised steps/meter *F* (3,464) = 17.5, p <0.001). In age-stratified groups, the difference in single-tasking walks became less pronounced (50-to-70y UP: Steps/meter *F* (3,66) = 6.44, p <0.001); normalised steps/meter *F* (3,68) = 3.41, p = 0.02, respectively; FP: Steps/meter *F* (3,68) = 3.86, p = 0.01; normalised steps/meter *F* (3,68) = 3.45, p = 0.02; SP: Steps/meter (*F* (3,25) = 2.61, p>0.05; normalised steps/meter *F* (3,67) = 0.67, p>0.05; 70-to-80y UP: Steps/meter *F* (3,180) = 6.29, p <0.001); normalised steps/meter *F* (3,218) = 5.21, p = 0.002, respectively; FP: Steps/meter *F* (3,207) = 2.28, p>0.05; normalised steps/meter *F* (3,217) = 3.33, p = 0.02; SP: Steps/meter(*F* (3,48) = 5.52, p = 0.003; normalised steps/meter *F* (3,218) = 2.95, p = 0.03); over 80y: UP: Steps/meter *F* (3,130) = 3.69, p = 0.01); normalised steps/meter *F* (3,223) = 2.76, p = 0.04, respectively; FP: Steps/meter *F* (3,191) = 4.86, p = 0.003; normalised steps/meter *F* (3,217) = 2.56, p>0.05; SP: Steps/meter(*F* (3,26) = 0.14, p>0.05; normalised steps/meter *F* (3,208) = 1.15, p>0.05), while dual-tasking walks remained significant (50-to-70y AW: Steps/meter *F* (3,35) = 1.95, p>0.05; normalised steps/meter *F* (3,65) = 2.52, p>0.05; CW: Steps/meter *F* (3,37) = 3.97, p = 0.02; normalised steps/meter *F* (3,61) = 1.45, p>0.05; 70-to-80y AW: Steps/meter *F* (3,79) = 6.16, p <0.001; normalised steps/meter *F* (3,212) = 7.16, p <0.001; CW: Steps/meter *F* (3,80) = 5.35, p = 0.002; normalised steps/meter *F* (3,204) = 8.28, p <0.001; over 80y: AW: Steps/meter *F* (3,33) = 0.08, p>0.05; normalised steps/meter *F* (3,197) = 5.15, p = 0.002; CW: Steps/meter *F* (3,44) = 0.74, p>0.05; normalised steps/meter *F* (3,197) = 3.90, p = 0.01). Reduced step length (more steps per meter) was associated with cognitive impairment, especially in dual-tasking conditions. In the latter, significance also remained after age stratification).

Step width is a variable relating to gait instability, especially in counting dual tasking [36]. We found no significant difference in step width in either walks except for AW in the total group and no significance in the age-stratified groups older than 70 years.

The step width variability was no significant as a differentiator between cognitive groups.

Cycle time variability was not significantly different between the four cognitive stages except in the entire population SP-set up (*F* (3,101) = 3.50,p = 0.02).

Swing time variability increased as cognition decreased (real data in S5 Table). This association was significant in all walking conditions except SP in the entire group (UP *F* (3,379) = 4.85, p = 0.003; FP: *F* (3,466) = 7.90, p<0.001; SP: *F* (3,101) = 1.27, p>0.05; AW: *F* (3,149) = 7.90, p = 0.01; CW: *F* (3,163) = 4.18, p = 0.007). After age-stratification, none of the conditions remain significant.

DTC calculation AW versus UP (and not CW versus UP) for gait speed, its normalised value, mean step length (steps per meter) and swing time variability, were significantly different between cognitive stages in the entire study population (DTC gait speed *F* (3,483) = 2.86, p = 0.04, DTC normalised gait speed F (3,476) = 3.16, p = 0.02; DTC steps/meter *F* (3,480) = 6.20, p < 0.001, DTC Swing time variability *F* (3,149) = 5.30, p = 0.002). This difference remained detectable only in the age-matched participants older than age 80 years (DTC gait speed *F* (3,200) = 4.58, p = 0.004, DTC normalised gait speed F (3,197) = 4.88, p = 0.003; DTC steps/meter *F* (3,200) = 3.86, p = 0.01, DTC Swing time variability *F* (3,33) = 2.17, p>0.05)).

**Fig 2:**
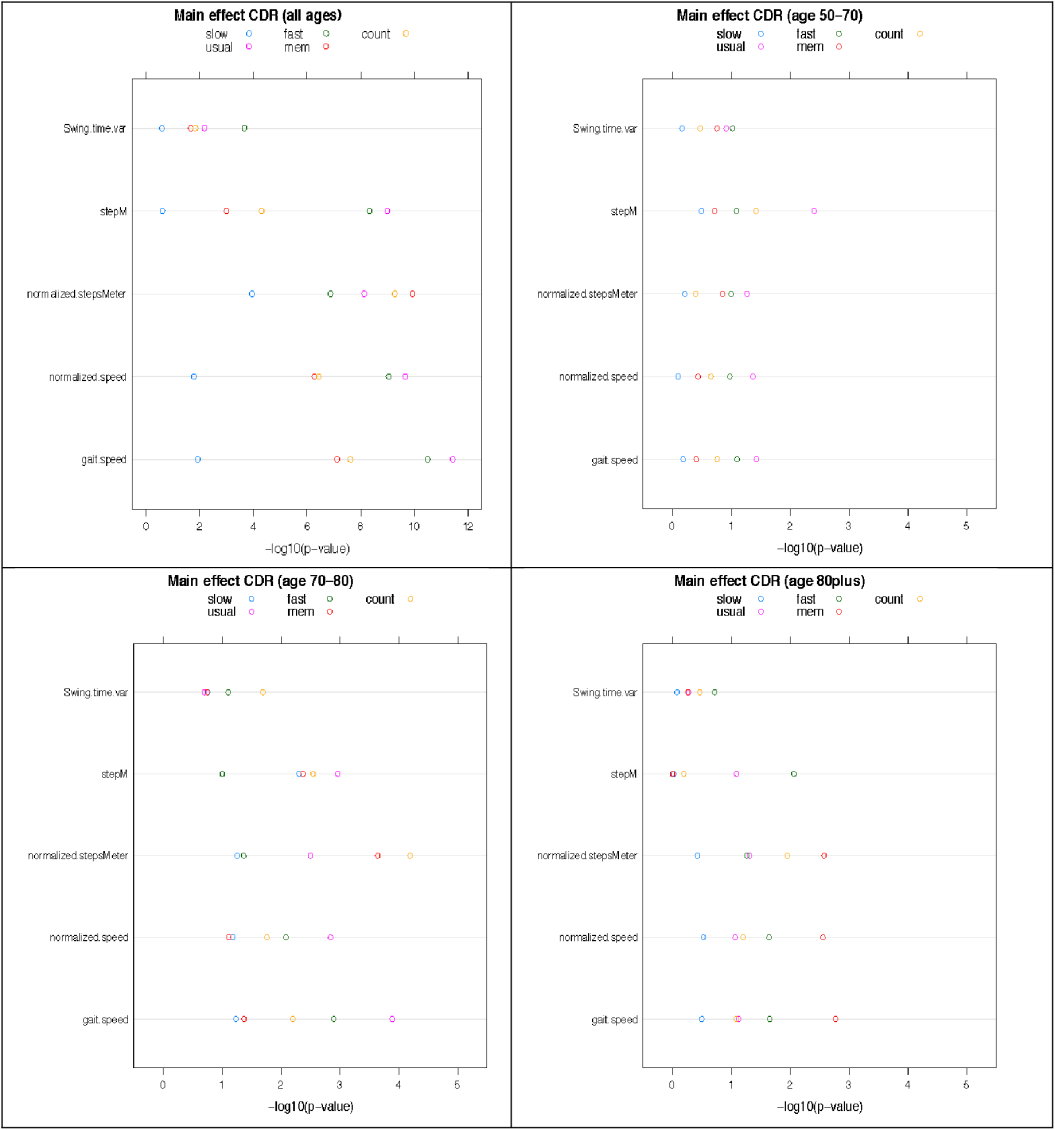
Dotplot: Multivariate regression model for association between dementia stages (CDR code) and gait variables adjusted for gender in five walking conditions for all participants and for age-stratified groups (two-way ANOVA). Dotplot_all: Main effect test of CDR on gait parameter, for all ages combined. A 2-way ANOVA model was fitted, with main effects for CDR score and gender but without the interaction term. The dotplot shows the negative logarithm (10-based) of the p-value for the main effect of CDR score.Dotplot_5070: Main effect test of CDR score on gait parameter (age 50 to 70). Dotplot_7080: Main effect test of CDR score on gait parameter (age 70–80). Dotplot_80plus: Main effect test of CDR score on gait parameter (above age 80).

We repeated the previous analyses using multiple linear regression analysis with adjustment for age and gender in the most significant parameters (Fig 2 and S3 Table).

This leads to very similar conclusions with an overall significant association between spatial-temporal gait variables after adjustment for age and gender and dementia stage in CDR code in the entire study group. Particularly gait speed, normalised gait speed, steps per meter and normalised steps per meter and swing time variability were stable parameters (p values all between 0.01 an <0.001 in UP, FP, AW, CW, not in SP)(*F* an p-values in the table in S3 Table). The selected spatial-temporal gait parameters as in the above remained significantly different between the age-matched groups especially in the UP, FP and AW walking condition and in the normalised steps/meter variable.

Interaction test between gender and CDR score to test if the difference in gait characteristics between CDR scores is the same in males and females showed for none of the gait characteristics a significant interaction between gender and CDR scores. Results are not reported.

### Predicting the probable presence of dementia or cognitive impairment combining spatial-temporal gait parameters using a probability model

To assess whether the gait parameters could be used to predict an individual’s cognitive status, we fitted logistic regression models. The first model predicted the probability for dementia. The second model predicted the probability for cognitive impairment (S4 Table). Separate models were constructed for each of the spatial-temporal gait parameters and walking conditions. Subsequently, Receiver Operating Characteristic (ROC) curves were constructed, and the Area Under the Curve (AUC) values calculated. Most AUC values were between 0.60 and 0.73 indicating that none of the separate gait parameters had a strong individual predictive power.

**Fig 3:**
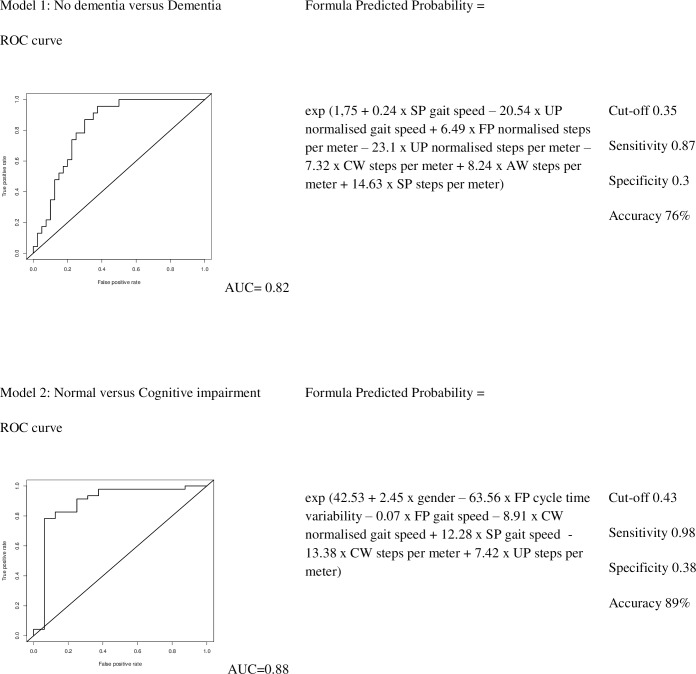
Multivariable logistic regression Models 1 and 2. ROC: receiver operating characteristic. AUC: area under the curve. Walking conditions: UP = Usual pace, FP = Fast pace, SP = Slow pace, CW = count walk, AW = animal walk.

To assess if the combination of several gait parameters could improve the prediction of cognitive status, we constructed multiple logistic regression models. Here too, the first set of models predicted the probability of having dementia for individual patients. This model is shown in Model 1 Formula (Fig 3). Using a predicted probability cut-off of 0.35, the model showed a sensitivity of 0.87, and a specificity of 0.30, with an overall 76% accuracy. Although age entered as a covariate in the starting model, this variable was not retained as a predictor in the final model. The AUC of this model was 0.82, as shown in Fig 3.

Multivariable logistic regression analysis of the second model, predicting the probability of being a cognitively impaired individual (Model 2 Formula, Fig 3) resulted in an equation with 89% accuracy, when a predicted probability cut-off of 0.43 was used with a sensitivity of 0.98 and a specificity of 0.38. Age was not retained as a predictor in the final model, but gender was. The AUC of this model was 0.89, as shown in Fig 3.

To test if the CDR score could be predicted based upon one or more spatial-temporal gait parameters, we fitted proportional odds models. However, none of the models could reliably predict the CDR scores. Even combinations of gait parameters or limiting the population to subtypes of dementia, did not improve the prediction of separate CDR scores. Full descriptive statistics for continuous variables expressed in mean values with standard deviations are available in the table in S5 Table.

## Discussion

This study provides insight into the additional value of using age-stratification, normalisation-for-leg-length and the selection of spatial-temporal gait characteristics in different walking conditions to predict cognitive impairment on an individual basis. Integrating these selected parameters in a model may distinguish patients with possible presence of cognitive impairment or dementia and suggest a new method useful in the cognitive diagnostic process in routine clinical practice.

### Age stratification

We observed that age-stratification revealed different associations between gait and cognition that were not detectable in the entire study group. Our results show that age influences gait performance and stability, demonstrated by the change spatial-temporal gait characteristics in different age groups. The values changed with age, but the difference between the cognitive stages was still detectable. This suggests that these parameters are influenced by both age and cognition. There are multiple underlying mechanisms like neuronal aging, balance or sarcopenia that could explain these differences. Firstly, the effect of neuronal ageing may play a role. Already in 1988 Schiffler described changes in the frontal lobe, extrapyramidal signs, changes to the posterior track of the spine and changes in peripheral nervous system in cognitively healthy older 80-plus subjects. All these changes may influence the spatial and temporal aspects of gait. We could assume that these changes are the primary explanation for difference between age groups. As our subjects deteriorate more in these regions in the central nervous system during dementia processes, this may have impact on their gait abilities. Furthermore, balance as a separate indicator may have an effect on gait performance. However, balance and stability testing in our data seemed less sensitive to cognitive changes, although the statistics on the entire group would suggest a relation. This result may be due to the small number of data in these tests. Thirdly, the effect of muscle mass and muscle function was not ruled out as a factor influencing gait preformance. Body composition is, to our knowledge, not described as a factor influencing cognitive changes. Van Kan et al [37,38] concluded in the EPIDOS-study that body composition changes are not related tot cognitive dysfunction. They concluded that there is no association between body composition changes or sarcopenia and cognitive dysfunction changes. This aspect remains still open for discussion and research.

Our study confirms that the functional performance and disability levels according to cognition evolved in the same manner in the different age groups as in the entire group. However, whereas most of the spatial-temporal gait characteristics differentiated cognitive impairment stages in the entire group, results after age-stratification was variable. Gait speed and normalised gait speed remained able to differentiate between the cognitive stages in all walking conditions in the age groups 70-to-80 and over-80. Younger participants were less differentiated. This was presumingly due to the lower power of these data due to a lower number of participants. Steps per meter (or mean step length) and normalised steps per meter differentiated cognitive groups in all conditions in the entire group, as well as when age groups were considered. More attention should be given to step length as a discriminative parameter. Counting steps and recalculating them in steps per meter seem a feasible method for general practitioners and need more research in this context. Dual task testing appears to be a stable indicator for cognitive differentiation in specific age groups. Notably, they only differentiate cognitive groups in over-80-year olds in AW dual tasking parameters; namely steps per meter, normalised steps per meter, swing time variability, step width and dual-task-cost for normalised gait speed. Step width variability in CW dual tasking differentiated only in 70-to-80 year olds. This suggests that when these gait parameters are considered for prediction profiling, dual tasking tests have only a differentiation potential when age is taken into account in a formula for predicting the cognitive stage of an individual.

### Normalisation of leg length

The effect of normalisation for leg length in gait speed and step length parameters could substitute for taking into account body height or even gender. To our knowledge, normalising gait speed and step length for leg length has not been studied in cognitively impaired people before. In our study, the application of this correction ameliorated the differentiation between stages of cognitive impairment in certain situations moreover when adjustment for gender was applied and confirmed our hypothesis that gait parameters corrected of leg-length are not influenced by gender. In the multivariate analysis adjusted for gender and age, we observed a limited added value of normalisation in the entire study cohort. When age stratification was applied, however, we detected a clearer association between the normalised parameters and cognitive impairment stages than the non-normalised parameters. The correction for leg length generates more statistically meaningful gait parameters for discrimination of cognitive impairment stages. Especially normalised step length has more power to differentiate between cognitive stages. This conclusion strengthens the assertion that normalisation for leg length may have an impact on gait-related research conclusions and is to be considered as study variables when age and gender are involved in the research objectives.

### Selection of spatial-temporal gait parameters

Previous studies indicate that gait speed, stride velocity, stride-time variability, stride length and the co-variation of stride length are related to dementia stages. They showed that the motor phenotype seem useful for screening and early diagnosis of cognitive impairment [6,10,35,39].Cedervall et al concluded in a longitudinal study on a small group of patients that gait speed, step length and the effect of dual tasking were important temporal and spatial gait changes indicating cognitive impairment and cognitive changes. Kikkert reported in a recent systematic review that using walking ability as a marker could improve the accuracy and specificity of neuropsychological testing and biological markers. They concluded that gait speed alone lacked specificity. However, dynamic gait parameters, like step frequency, variability and step length, included in an add-on gait analysis would improve the results.

Our study confirms that similar parameters; namely gait speed, mean number of steps per meter (similar to step or stride length) and swing time variability (equal to step time variability), were associated with the severity of cognitive impairment at UP. Gait speed decreased, number of steps swing time variability increases as cognitive impairment became more severe. Decreasing step length (increasing number of steps per meter) with progressive dementia has already been described in a longitudinal study on gait function in demented patients tested at UP [40]. We were able, however, to observe this phenomenon at earlier stages of cognitive impairment. Steps per meter, but also swing time variability, showed significant changes between normal, pre-dementia and dementia stages at UP. Thus, not only gait speed but also other spatial-temporal gait parameters change in pre-dementia stages and could be useful for predicting cognitive change. We speculate that these changes could be the result of early change in the brain structure that occurs in pre-stages of dementia. Sakurai et al. recently reported that the reduced step length and increased swing time variability could be related to reduced activity in the right posterior cingulate and primary sensorimotor cortices on functional MRI in older women, supporting this theory.

### Predictive capacities of gait parameters

Our hypothesis that the use of a standardised multiple-walking-condition-test-configuration increases the number of eligible differentiation variables and predictive abilities is supported by our results. In addition to usual pace parameters, other parameters in other walking conditions were shown to be useful for differentiation.

Previous studies in impaired cognition confirmed that walking condition, other than usual pace, influenced gait. For instance, dual tasking or fast gait had implications for the variability of step time and stride time in people with MCI [41–43]. This indicates that a test setup with multiple test conditions would broaden diagnostic opportunities.

Our study designed five walking conditions based on the test modes used in the literature for MCI detection, the first clinical presentation of cognitive decline. Using this method, we were able to expand the possible attributes in gait analysis and the selectable parameters for the detection of cognitive impairment. We identified that variables in other walking conditions (FP, SP, AW and CW) were also related to the change in cognitive impairment stage. This implies that the discriminative power of a gait parameter can be established in various walking conditions and not only at usual pace. However, our results confirm that single parameters are not powerful enough to predict the cognitive status. More than one parameter in a single test mode is needed to uncover cognitive changes. Therefore, we created two models predicting the presence dementia or assuming cognitive impairment on an individual basis. The model formulas are combinations of the most sensitive parameters in the multi-testing procedure. These predictive probability formulas have an acceptable sensitivity and accuracy to indicate the possible presence of dementia or cognitive impairment. The formulas contain parameters identified in previous studies, but also the new variables, normalised gait speed, step per meter and dual task parameters, illustrating the additional value of multi-testing and normalisation for leg length. The adjustment for age seems not to be necessary when these combinations are applied. The models confirm the found by Deshpande [44]. Furthermore, these models confirm the relevance of a combination of variables. These results are to be considered meaningful because defining the most significant gait characteristics in multiple test settings creates a collection of parameters with more weight on detecting cognitive decline. One single parameter of this collection is not powerful enough to distinguish the cognitive stages. However, a compiled model may be more conclusive. These models are integrable in the electronic walkway system software and open possibilities for more clinical use. The steps-per-meter parameter is also auspicious. This result suggests that counting the footsteps over a fixed distance in a walking condition appropriate for the age group examined in clinical practice would be able to suspect cognitive decline.

The validation of our models in the future could make structured gait analysis applicable in a diagnostic pathway, and improves the referral of individual patients for further examination in a clinically feasible manner. The models could be fitted in the computerised gait system and make the method more user-friendly.

The power of our research includes the single centre cohort and single investigator set-up, the size and the comprehensive cognitive and geriatric assessment on a well-defined population. The test environment and test conditions were standardised. The assessment tools were identical in all participants. The added value of normalisations for leg length in gait analysis provides innovation for future gait research.

Some limitations of this study need to be discussed. The strict exclusion criteria of age, language and ethnicity specifying the test group to an aged Caucasian population may hinder extrapolation to other ethnic groups. The diagnoses were made on a clinical basis and not confirmed anatomopathologically and the cross sectional and retrospective set-up limits the concurrent validity of the findings. This is a single centre cohort and single investigator set-up study, which may have introduced bias. We acknowledge a selection bias because the study was performed in an ambulatory memory clinic. We reached the subjects that were referred by a general practitioner, a relative or a community social worker. We did not reach persons without cognitive complaints; people living alone without informal or formal care givers, and physically frail persons. In addition, excluding pre-existing gait problems, severe physical frailty and severe dementia implied a higher success rate of performing the five walking conditions during multi-testing. Including these groups, however, would have implied a higher risk of drop out during the five-walk-test and registration of constant slow walks. Since this study focused on early detection, documenting gait patterns in stages of severe dementia would have no added value for our research question. Furthermore, in previous studies walking too slow was considered to be related to cognitive decline and had no added value for discriminative power [6,35] Our study results support these findings, making clear that slow pace was less usable to discriminate between cognitive stages.

Due to the retrospective and cross-sectional study composition, validation of the predictive probability formulas is still necessary before they can be used in routine clinical care as an assessment or screening tool. Although the accuracy and sensitivity of the models was relatively high, the specificity was rather low. This implies that these models are not usable as stand-alone diagnostic tests, but are more interesting as screening procedures. Also in this phase of the study, only the spectrum of cognitive impairment is discussed and dementia sub-typing is still to be studied.

Future research is needed to validate the compilation models as potential assessment tools. Future work should include prospective, longitudinal data analysis and differentiation of dementia types with gait analysis.

## Conclusions

Our study aimed to clarify the association between standard clinical cognitive classification and a multiple walking condition test mode in order to gain insight in the possibilities of building motor evaluation into cognitive screening for geriatric community-dwelling people. We also sought to expand the number of selectable gait variables to broaden the ability of gait cycle for screening and detecting cognitive impairment. We found that no single gait variable, but rather a combination of different gait variables selected from multiple walking conditions, could be integrated into two differentiating formulas. These formulas could separate dementia patients from those without dementia or separate normal individuals from those with cognitive impairment. The standardised five walking conditions computerised gait analysis is an accessible method that may improve the current standard testing for dementia. This study provides new insight for future work. The findings can be used to develop a uniform test protocol for routine clinical screening to differentiate dementia stages when memory complaints start to occur.

## Supporting information

S1 Table Association of clinical characteristics in the total study population and age-stratified groups in four different dementia stages (one-way ANOVA and chi-square test). (PDF)

S2 Table Association between dementia stage (CDR code) and gait variables (one-way ANOVA). (PDF)

S3 Table Multivariate regression model for association between dementia stage (CDR code) and gait variables adjusted for gender in five walking conditions for all participants and for age-stratified groups (two-way ANOVA). (PDF)

S4 Table Simple logistic regression analysis for Model 1 and 2. (PDF)

S5 Table Real data descriptive statistics. (PDF)
