# Supplementary material for: Gait characteristics under different walking conditions: Association with the presence of cognitive impairment in community-dwelling older people
Source: PLoS One. 2017 Jun 1;12(6):e0178566. doi: 10.1371/journal.pone.0178566 (PMC5453541; doi:10.1371/journal.pone.0178566)
Supplement: S1 Table — (PDF) [file pone.0178566.s001.pdf]

Table 1. Association of clinical characteristics in the total study population and age-stratified groups in four different dementia stages (One-way ANOVA and Chi-square test)

		Dementia stages				Test statistics
CDR group		CHI	MCI	Mild	Moderate	
Variables	Units					
**Total study population**						
Number of participants	n	78	140	222	96	Total N = 536
Age	years	75.0±8.9	79.0±7.2	81.0 ± 7.4	83.0 ± 6.8	*F* (3,533) = 16.08, p **< 0.001**
Gender Male	%	46	37	35	26	*X^2 (3, N = 536) = 7.89,* ***p = 0.05***
Education	years	12.0±2.6	12.0±3.1	10.0 ± 2.5	9.0 ± 2.4	*F* (3,533) = 16.07, p **< 0.001**
MMSE		29.0 ± 0.7	26.0±0.8	22.0 ± 1.6	15.0 ± 2.9	*F* (3,532) = 1250.25, p **< 0.001**
ACE-R		81.0 ± 8.7	71.0±7.7	58.0 ± 8.7	38.0 ± 11.8	*F* (3,473) = 314.85, p **< 0.001**
NPI-Q		5.0±9.3	8.0±14.0	11.0 ± 13.2	14.0 ± 13.6	*F* (3,433) = 5.16, p **= 0.002**
Depression	%	44	46	37	35	X^2 (3, N = 536) = 4.25, p > 0.05
Diabetes	%	10	26	12	10	X^2 (3, N = 536) = 15.52, **p =0.001**
Hypertension	%	32	44	46	42	X^2 (3, N = 536) = 5.13, p > 0.05
COLD	%	9	9	7	7	X^2 (3, N = 536) = 0.93, p > 0.05
Falls	%	13	19	25	15	X^2 (3, N = 536) = 7.95, ***p = 0.05***
TGUG	sec	8.0± 2.8	11.5±6.4	10.0 ± 3.6	14.0 ± 7.0	*F* (3,67) = 3.94*,* ***p = 0.01***
TCST	sec	14.0 ± 3.5	14.0±7.7	16.0 ± 5.9	18.0 ± 6.2	*F* (3,196) = 2.49*,* ***p = 0.06***
FR	sec	26.0 ± 8.3	24.0±11.3	26.0 ± 6.2	22.0 ± 5.5	*F* (3,24) = 1.28*,* p > 0.05
Katz		6.0 ± 0.9	6.0±1.9	6.0 ± 1.2	5.0 ± 1.5	*F* (3,488) = 11.99*,* **p < 0.001**
Lawton		6.0 ± 1.9	6.0 ±2.3	4.0 ± 2.6	2.5 ± 2.3	*F* (3,410) = 28.37*,* **p < 0.001**
Rockwood		4.0 ± 1.2	4.0±1.0	5.0 ± 1.0	5.0 ± 0.8	*F* (3,507) = 35.90, p **< 0.001**
NumMed		4.5 ± 3.1	5.0±3.3	5.0 ± 3.3	4.0 ± 2.8	*F* (3,452) = 1.94*,* p > 0.05
**50 to 70 years old participants**						

Number of participants	n	20	21	24	6	Total N = 71
Age	years	61.8 ± 5.8	64.3±4.1	64.8 ± 3.5	66.7 ± 2.7	*F (3,67) = 2.26,* ***p =0.09***
Gender Male	%	65	43	58	17	X^2 (3, N = 71) = 1.21, p > 0.05
Education	years	12.6 ± 2.3	12.3±3.8	11.0 ± 2.9	11.3 ± 1.1	*F (3,67) = 7.09,* **p < 0.001**
MMSE		29.0 ± 0.5	26.1±0.8	22.3 ± 1.5	15.2 ± 2.1	*F (3,67) = 195.72,* **p < 0.001**
ACE-R		85.6 ± 7.0	71.6 ±7.8	61.5 ± 10.0	46.6 ± 19.1	*F (3,62) = 37.85***, p < 0.001**
NPI-Q		7.0 ± 9.5	20.0±15.4	15.0 ± 11.8	13.3 ± 14.8	*F (3,56) = 3.48,* **p = 0.02**
Depression	%	40	57	42	50	X^2 (3, N = 71) = 1.55, p > 0.05
Diabetes	%	0	19	8	0	X^2 (3, N = 71) = 6.91, ***p =0.07***
Hypertension	%	10	29	29	67	X^2 (3, N = 71) = 7.73, ***p = 0.05***
COLD	%	5	19	0	0	X^2 (3, N = 71) = 7.78, ***p = 0.05***
Falls	%	10	4	2	NaN	X^2 (3, N = 71) = 1.25, p > 0.05
TGUG	sec	8.0 ± 1.0	8.0±1.4	9.0 ± 2.8	NaN	*F (2,8) = 0.36,* p > 0.05
TCST	sec	11.5 ± 3.9	15.5±3.6	12.0 ± 4.8	NaN	*F (2,25) = 1.45,* p > 0.05
FR	sec	32.0 ± 8.5	47.0±10.8	27.0 ± 1.41	NaN	*F (2,7) = 2.05,* p > 0.05
Katz		6.0 ± 0.2	6.0 ±1.0	6.0 ± 0.5	4.0 ± 1.5	*F (3,64) = 2.02***,** p > 0.05
Lawton		6.5 ± 1.8	5.4±2.8	5.7 ± 2.0	2.3 ± 2.1	*F (3,55) = 5.79,* **p < 0.001**
Rockwood		3.3 ± 1.3	4.0±0.9	4.2 ± 0.9	4.7 ± 1.1	*F (3,66) = 4.6,* p **< 0.006**
NumMed		2.7 ± 0.8	3.5±0.7	4.0 ± 0.7	2.6 ± 1.5	*F (3,55) = 0.57,* p > 0.05

70 to 80 years old participants

Number of participants	n	42	75	87	34	Total N = 238
Age	years	75.0 ± 2.9	75.5±2.8	75.0 ± 2.8	75. ± 6.6	*F (3,234) = 0.72,* p > 0.05
Gender Male	%	43	39	29	32	X^2 (3, N = 238) = 3.20, p > 0.05
Education	years	12.0 ± 2.9	12.0±2.4	11.0 ± 2.7	10.0 ± 2.8	*F (3,234) = 7.09,* **p < 0.001**
MMSE		28.0 ± 0.8	26.0±0.8	22.0 ± 1.6	15.0 ± 3.4	*F (3,234) = 504.71,* **p < 0.001**

ACE-R		80.0 ± 7.6	72.0±8.6	60.0 ± 8.2	38.0 ± 11.4	*F (3,216) = 168.21*, **p < 0.001**
NPI-Q		4.0 ± 8.5	4.0±14.1	10.0 ± 13.9	15.0 ± 16.5	*F (3,185) = 4.02*, **p = 0.008**
Depression	%	51	35	37	45	X^2 (3, N = 238) = 1.37, p > 0.05
Diabetes	%	16	28	17	10	X^2 (3, N = 238) = 4.84, p > 0.05
Hypertension	%	35	48	38	48	X^2 (3, N = 238) = 1.60, p > 0.05
COLD	%	11	8	11	10	X^2 (3, N = 238) = 0.39, p > 0.05
Falls	%	16	22	21	14	X^2 (3, N = 238) = 3.20, p > 0.05
TGUG	sec	8.0 ± 2.6	11.5±1.9	9.0 ± 2.2	14.0 ± 5.0	*F (3,21) = 2.52,* ***p =0.08***
TCST	sec	14.0 ± 2.6	14.0±5.6	15.0 ± 4.7	18.0 ± 7.0	*F (3,81) = 1.01,* p > 0.05
FR	sec	26.0 ± 6.4	26.0±7.2	27.0 ± 6.5	23.5 ± 5.5	*F (3,23) = 1.45,* p > 0.05
Katz		6.0 ± 0.7	6.0 ±0.9	6.0 ± 1.0	6.0 ± 1.4	*F (3,213) = 4.35*, **p = 0.005**
Lawton		6.0 ± 1.8	6.0±2.3	5.0 ± 2.5	3.0 ± 2.2	*F (3,181) = 9.72,* **p < 0.001**
Rockwood		4.0 ± 1.1	4.0±1.1	5.0 ± 0.9	5.0 ± 0.8	*F (3,221) = 15.1,* p **< 0.001**
NumMed		5.0 ± 3.6	5.0±3.6	4.0 ± 3.7	4.0 ± 2.6	*F (3,199) = 0.79,* p > 0.05

>80 years old participants

Number of participants	n	23	62	130	63	Total N= 278
Age	years	83.0 ± 2.8	83.0±2.8	84.0 + 3.5	85.0 ± 3.6	*F (3,274) = 5.12,* ***p*** **= 0.002**
Gender Male	%	41	11	32	24	X^2 (3, N = 278) = 2.43, p > 0.05
Education	years	12.0 ± 2.3	12.0±3.8	10.0 ± 2.2	8.0 ± 2.2	*F (3,274) = 5.82,* ***p*** **< 0.001**
MMSE		29.0 ± 0.8	26.0±0.8	21.5 ± 1.6	15.0 ± 2.8	*F (3,273) = 570.40,* ***p*** **< 0.001**
ACE-R		78.5 ± 9.9	69.0±5.9	55.0 ± 8.3	38.0 ± 12.6	*F (3,235) = 126.90,* ***p*** **< 0.001**
NPI-Q		7.0 ± 10.4	8.5±12.6	10.0 ± 13.1	14.0 ± 12.5	*F (3,225) = 1.19,* p > 0.05
Depression	%	32	52	37	30	X^2 (3, N = 278) = 7.06, p > 0.05
Diabetes	%	9	27	10	11	X^2 (3, N = 278) = 10.55, ***p = 0.01***
Hypertension	%	50	45	53	43	X^2 (3, N = 278) = 2.18, p > 0.05
COLD	%	9	8	5	09	X^2 (3, N = 278) = 0.95, p > 0.05

Falls	%	14	21	31	16	X^2 (3, N = 278) = 7.19, ***p = 0.07***
TGUG	sec	13.0 ± 4.2	13.0±7.3	11.0 ± 3.8	13.5 ± 8.2	*F (3,33) = 1.16,* p > 0.05
TCST	sec	16.9 ± 3.2	15.0±9.9	18.0 ± 6.3	17.0 ± 6.0	*F (3,99) = 0.26, p> 0.05*
FR	sec	18.5 ± 3.5	18.0±7.9	25.0 ± 6.0	20.0 ± 6.2	*F (3,31) = 1.08, p> 0.05*
Katz		6.0 ± 1.3	6.0±1.2	5.0 ± 1.3	5.0 ± 1.5	*F (3,252) = 4.66,* ***p* = 0.003**
Lawton		5.0 ± 2.0	5.0±2.0	3.0 ± 2.5	2.0 ± 2.2	*F (3,208) = 9.22,* ***p* < 0.001**
Rockwood		4.0 ± 1.2	4.0±0.9	5.0 ± 1.0	5.0 ± 0.8	*F (3,262) = 12.67,* ***p* < 0.001**
NumMed		5.0 ± 2.1	5.0±2.9	6.0 ± 3.0	4.0 ± 2.7	*F (3,238) = 3.05,* ***p = 0.03***

CDR: clinical dementia rating, CHI: cognitively healthy individuals = CDR 0, MCI : mild cognitive impaired = CDR 0.5, Mild : mild dementia = CDR1, Advanced : Advanced dementia = CDR 2 and 3, n = number MMSE : mini mental state evaluation, ACE-R : Addenbrooks' cognitive evaluation revised, NPI-Q : neuropsychological inventory – questionnaire, COPD: chronic obstructive pulmonary disease, TGUG : time get up and go, TCST : timed chair stands test , FR : functional reach, NumMed : number of medications , Significant p-values in bold, NaN= No available Number. Test statistics One -way ANOVA F (df1,df2) = y, p-value, Chi-square test X^2(df, N = x)= y, p-value)
