# Supplementary material for: Gait characteristics under different walking conditions: Association with the presence of cognitive impairment in community-dwelling older people
Source: PLoS One. 2017 Jun 1;12(6):e0178566. doi: 10.1371/journal.pone.0178566 (PMC5453541; doi:10.1371/journal.pone.0178566)
Supplement: S2 Table — (PDF) [file pone.0178566.s002.pdf]

Table 2. Association between dementia stage (CDR code) and gait variables (One-way ANOVA)

All participants	SP	UP	FP	AW	CW
**Normalized gait speed**					
p-value	**0.01**	**< 0.001**	**< 0.001**	**< 0.001**	**< 0.001**
F-value	3.85	18.5	17.4	12.1	12.4
df1	3	3	3	3	3
df2	499	514	504	477	465
**Gait speed**					
p-value	**0.004**	**< 0.001**	**< 0.001**	**< 0.001**	**< 0.001**
F-value	4.5	22.3	19.9	14.2	15.0
df1	3	3	3	3	3
df2	503	521	510	483	473
**Cadence**					
p-value	**0.02**	**0.02**	**0.04**	> 0.05	> 0.05
F-value	3.33	3.42	2.75	0.65	1.09
df1	3	3	3	3	3
df2	503	521	510	482	473
**Base of support**					
p-value	> 0.05	> 0.05	> 0.05	**0.003**	> 0.05
F-value	0.65	2.20	0.50	4.61	1.26
df1	3	3	3	3	3
df2	503	521	510	482	473
**Base of support variability**					
p-value	> 0.05	> 0.05	> 0.05	> 0.05	> 0.05
F-value	0.76	1.39	1.61	1.02	0.55
df1	3	3	3	3	3
df2	101	379	468	149	163
**Cycle time variability**					
p-value	**0.02**	> 0.05	> 0.05	> 0.05	> 0.05
F-value	3.50	1.62	0.98	1.03	1.57
df1	3	3	3	3	3
df2	101	379	466	149	162
**Swing time variability**					
p-value	> 0.05	**0.003**	**< 0.001**	**0.01**	**0.007**
F-value	1,27	4.85	7.90	3.92	4.18
df1	3	3	3	3	3
df2	101	379	466	149	163
**Steps/Meter**					
p-value	> 0.05	**< 0.001**	**< 0.001**	**< 0.001**	**< 0.001**
F-value	1.32	18.0	15.5	6.38	8.00
df1	3	3	3	3	3
df2	101	378	468	149	163
**DTC gait speed**					
p-value				**0.04**	> 0.05
F-value				2.86	1.05
df1				3	3
df2				483	472

DTC cadence					
p-value				> 0.05	> 0.05
F-value				0.72	0.81
df1				3	3
df2				482	472
DTC bass of support					
p-value				0.05	> 0.05
F-value				2.63	1.15
df1				3	3
df2				482	471
DTC bass of support variability					
p-value				> 0.05	> 0.05
F-value				0.43	0.69
df1				3	3
df2				147	162
DTC cylce time variability					
p-value				> 0.05	> 0.05
F-value				0.56	1.19
df1				3	3
df2				149	162
DTC swing time variability					
p-value				**0.002**	> 0.05
F-value				5.30	0.37
df1				3	3
df2				149	162
DTC steps/Meter					
p-value				**< 0.001**	**0.01**
F-value				6.20	3.77
df1				3	3
df2				480	470
DTC normalized gait speed					
p-value				**0.02**	> 0.05
F-value				3.16	0.79
df1				3	3
df2				476	465
Normalized steps/Meter					
p-value	**< 0.001**	**< 0.001**	**< 0.001**	**< 0.001**	**< 0.001**
F-value	8.03	16.0	13.4	19.1	17.5
df1	3	3	3	3	3
df2	495	511	502	476	464

50 to 70 years old participants					
	SP	UP	FP	AW	CW
Normalized gait speed					
p-value	> 0.05	**0.03**	**0.03**	> 0.05	> 0.05
F-value	0.34	3.10	3.14	0.88	1.49
df1	3	3	3	3	3
df2	68	68	68	66	61
Gait speed					

p-value	> 0.05	**0.02**	**0.02**	> 0.05	> 0.05
F-value	0.55	3.62	3.71	0.89	1.82
df1	3	3	3	3	3
df2	67	68	68	66	62
Cadence					
p-value	> 0.05	> 0.05	> 0.05	> 0.05	> 0.05
F-value	1.93	0.37	0.29	0.58	0.84
df1	3	3	3	3	3
df2	67	68	68	65	62
Base of support					
p-value	**0.05**	**0.04**	**0.006**	> 0.05	> 0.05
F-value	2.71	2.91	4.46	1.22	2.00
df1	3	3	3	3	3
df2	67	68	68	65	62
Base of support variability					
p-value	> 0.05	> 0.05	> 0.05	> 0.05	> 0.05
F-value	2.63	1.94	1.00	1.34	0.99
df1	3	3	3	3	3
df2	25	66	68	35	37
Cycle time variability					
p-value	> 0.05	> 0.05	> 0.05	> 0.05	> 0.05
F-value	2.90	0.01	0.17	0.93	0.64
df1	3	3	3	3	3
df2	25	66	68	35	37
Swing time variability					
p-value	> 0.05	> 0.05	**0.03**	> 0.05	> 0.05
F-value	0.44	2.02	3.06	1.87	1.54
df1	3	3	3	3	3
df2	25	66	68	35	37
Steps/Meter					
p-value	> 0.05	**< 0.001**	**0.01**	> 0.05	**0.02**
F-value	2.61	6.44	3.86	1.95	3.97
df1	3	3	3	3	3
df2	25	66	68	35	37
DTC gait speed					
p-value				> 0.05	> 0.05
F-value				0.11	0.29
df1				3	3
df2				66	62
DTC cadence					
p-value				> 0.05	> 0.05
F-value				0.51	0.88
df1				3	3
df2				65	62
DTC bass of support					
p-value				> 0.05	> 0.05
F-value				0.26	0.27
df1				3	3
df2				65	62
DTC bass of support					

variability					
p-value				> 0.05	> 0.05
F-value				0.69	0.67
df1				3	3
df2				35	36
DTC cylce time variability					
p-value				> 0.05	> 0.05
F-value				0.93	0.74
df1				3	3
df2				35	37
DTC swing time variability					
p-value				> 0.05	> 0.05
F-value				0.63	1.21
df1				3	3
df2				35	37
DTC steps/Meter					
p-value				> 0.05	> 0.05
F-value				1.34	1.58
df1				3	3
df2				65	62
DTC normalized gait speed					
p-value				> 0.05	> 0.05
F-value				0.11	0.05
df1				3	3
df2				66	62
Normalized steps/Meter					
p-value	> 0.05	**0.02**	**0.02**	> 0.05	> 0.05
F-value	0.67	3.41	3.45	2.52	1.45
df1	3	3	3	3	3
df2	67	68	68	65	61

70 to 80 years old participants

	SP	UP	FP	AW	CW
Normalized gait speed					
p-value	**0.05**	**< 0.001**	**0.004**	**0.05**	**0.01**
F-value	2.69	5.81	4.49	2.62	3.93
df1	3	3	3	3	3
df2	218	218	217	212	204
Gait speed					
p-value	**0.03**	**< 0.001**	**< 0.001**	**0.02**	**0.002**
F-value	2.97	8.07	6.09	3.30	4.99
df1	3	3	3	3	3
df2	221	222	220	215	207
Cadence					
p-value	> 0.05	> 0.05	> 0.05	> 0.05	> 0.05
F-value	1.08	1.47	2.19	0.47	0.35
df1	3	3	3	3	3
df2	221	222	220	215	207
Base of support					
p-value	> 0.05	> 0.05	> 0.05	> 0.05	> 0.05
F-value	0.58	0.71	0.28	1.17	0.68

df1	3	3	3	3	3
df2	221	222	220	215	207
Base of support variability					
p-value	> 0.05	> 0.05	> 0.05	> 0.05	> 0.05
F-value	0.20	1.08	1.92	0.36	0,40
df1	3	3	3	3	3
df2	48	181	207	79	80
Cycle time variability					
p-value	> 0.05	> 0.05	> 0.05	> 0.05	> 0.05
F-value	2.58	0.21	0.93	1.16	2.04
df1	3	3	3	3	3
df2	48	181	207	79	79
Swing time variability					
p-value	> 0.05	> 0.05	> 0.05	> 0.05	**0.01**
F-value	1.67	1.81	2.07	2.21	3.78
df1	3	3	3	3	3
df2	48	181	207	79	80
Steps/Meter					
p-value	**0.003**	**< 0.001**	> 0.05	**< 0.001**	**0.002**
F-value	5.52	6.29	2.28	6.16	5.35
df1	3	3	3	3	3
df2	48	180	207	79	80
DTC gait speed					
p-value				> 0.05	> 0.05
F-value				0.62	0.15
df1				3	3
df2				215	207
DTC cadence					
p-value				> 0.05	> 0.05
F-value				0.96	0.44
df1				3	3
df2				215	207
DTC bass of support					
p-value				> 0.05	> 0.05
F-value				0.45	1.19
df1				3	3
df2				215	207
DTC bass of support variability					
p-value				> 0.05	> 0.05
F-value				0.04	1.20
df1				3	3
df2				78	80
DTC cylce time variability					
p-value				> 0.05	> 0.05
F-value				0.77	2.17
df1				3	3
df2				79	79
DTC swing time variability					
p-value				**0.03**	> 0.05
F-value				3.11	0.82

df1				3	3
df2				79	79
DTC steps/Meter					
p-value				> 0.05	> 0.05
F-value				1.68	1.51
df1				3	3
df2				213	206
DTC normalized gait speed					
p-value				> 0.05	> 0.05
F-value				0.82	0.19
df1				3	3
df2				211	203
Normalized steps/Meter					
p-value	**0.03**	**0.002**	**0,02**	**< 0.001**	**< 0.001**
F-value	2.95	5.21	3.33	7.16	8.28
df1	3	3	3	3	3
df2	218	218	217	212	204

Over 80 years old participants

	SP	UP	FP	AW	CW
Normalized gait speed					
p-value	> 0.05	> 0.05	**0.03**	**0.003**	**0.05**
F-value	1.29	2.37	3.11	4.93	2.59
df1	3	3	3	3	3
df2	211	226	217E	197	198
Gait speed					
p-value	> 0.05	> 0.05	**0.03**	**0.001**	> 0.05
F-value	1.31	2.51	3.06	5.56	2.46
df1	3	3	3	3	3
df2	213	229	220	200	202
Cadence					
p-value	> 0.05	> 0.05	> 0.05	> 0.05	> 0.05
F-value	2.22	1.06	1.22	0.92	0.35
df1	3	3	3	3	3
df2	213	229	220	200	202
Base of support					
p-value	> 0.05	> 0.05	> 0.05	> 0.05	> 0.05
F-value	0.51	0.85	0.25	1.22	0.25
df1	3	3	3	3	3
df2	213	229	220	200	202
Base of support variability					
p-value	> 0.05	> 0.05	> 0.05	> 0.05	> 0.05
F-value	1.10	1.90	0.70	0.72	1.42
df1	3	3	3	3	3
df2	26	130	191	33	44
Cycle time variability					
p-value	> 0.05	**0.03**	> 0.05	> 0.05	> 0.05
F-value	0.40	3.12	1.03	2.15	0.89
df1	3	3	3	3	3
df2	26	130	189	33	44

Swing time variability					
p-value	> 0.05	> 0.05	> 0.05	> 0.05	> 0.05
F-value	0.48	0.89	1.86	0.83	1.05
df1	3	3	3	3	3
df2	26	130	189	33	44
Steps/Meter					
p-value	> 0.05	**0.01**	**0.003**	> 0.05	> 0.05
F-value	0.14	3.69	4.86	0.08	0.74
df1	3	3	3	3	3
df2	26	130	191	33	44
DTC gait speed					
p-value				**0.004**	> 0.05
F-value				4.58	1.85
df1				3	3
df2				200	201
DTC cadence					
p-value				> 0.05	> 0.05
F-value				1.98	1.42
df1				3	3
df2				200	201
DTC bass of support					
p-value				> 0.05	> 0.05
F-value				1.98	0.66
df1				3	3
df2				200	200
DTC bass of support variability					
p-value				> 0.05	> 0.05
F-value				0.65	0.53
df1				3	3
df2				32	44
DTC cylce time variability					
p-value				> 0.05	> 0.05
F-value				2.25	1.09
df1				3	3
df2				33	44
DTC swing time variability					
p-value				> 0.05	> 0.05
F-value				2.17	0.17
df1				3	3
df2				33	44
DTC steps/Meter					
p-value				**0.01**	> 0.05
F-value				3.86	2.31
df1				3	3
df2				200	200
DTC normalized gait speed					
p-value				**0.003**	> 0.05
F-value				4.88	1.84
df1				3	3
df2				197	198

Normalized steps/Meter					
p-value	> 0.05	**0.04**	> 0.05	**0.002**	**0.01**
F-value	1.15	2.76	2.56	5.15	3.90
df1	3	3	3	3	3
df2	208	223	215	197	197

CDR: Clinical Dementia rating. DTC : dual task cost. UP: Usual pace, FP: Fast pace, SP: slow pace, AW: animal reciting walk, CW: counting walk. p value significant when ≤0.05 (in bold).
