# Supplementary material for: Gait characteristics under different walking conditions: Association with the presence of cognitive impairment in community-dwelling older people
Source: PLoS One. 2017 Jun 1;12(6):e0178566. doi: 10.1371/journal.pone.0178566 (PMC5453541; doi:10.1371/journal.pone.0178566)
Supplement: S3 Table — (PDF) [file pone.0178566.s003.pdf]

Table 3. Multivariate regression model for association between dementia stage (CDR code) and gait variables adjusted for gender in five walking conditions for all participants and for age-stratified groups (two-way ANOVA)

All participants

	SP	UP	FP	AW	CW
Gait speed					
p-value	**0.01**	**< 0.001**	**< 0.001**	**< 0.001**	**< 0.001**
F-value	4	19.8	18.2	12.4	13.3
df1	3	3	3	3	3
df2	502	520	509	482	472
Normalized gait speed					
p-value	**0.02**	**< 0.001**	**< 0.001**	**< 0.001**	**< 0.001**
F-value	3	16.8	15.7	11.0	11.3
df1	3	3	3	3	3
df2	498	513	503	476	464
Steps/Meter					
p-value	> 0.05	**< 0.001**	**< 0.001**	**0.001**	**< 0.001**
F-value	1	15.9	14.5	5.71	8.06
df1	3	3	3	3	3
df2	100	377	467	148	162
Normalized steps/Meter					
p-value	**< 0.001**	**< 0.001**	**< 0.001**	**< 0.001**	**< 0.001**
F-value	7	14.1	12.0	17.3	16.2
df1	3	3	3	3	3
df2	494	510	501	475	463
Swing time variability					
p-value	> 0.05	**0.007**	**< 0.001**	**0.02**	**0.01**
F-value	1	4.14	6.64	3	4
df1	3	3	3	3	3
df2	100	378	465	148	162

50 to 70 years old participants

	SP	UP	FP	AW	CW
Gait speed					
p-value	> 0.05	**0.04**	> 0.05	> 0.05	> 0.05
F-value	0.54	2.99	2.37	1.02	1.71
df1	3	3	3	3	3
df2	66	67	67	65	61
Normalized gait speed					

p-value	> 0.05	**0.04**	> 0.05	> 0.05	> 0.05
F-value	0.33	2.88	2.12	1.08	1.52
df1	3	3	3	3	3
df2	67	67	67	65	60
Steps/Meter					
p-value	> 0.05	**0.004**	> 0.05	> 0.05	0.04
F-value	1.25	4.94	2.35	1.69	3.15
df1	3	3	3	3	3
df2	24	65	67	34	36
Normalized steps/Meter					
p-value	> 0.05	**0.05**	> 0.05	> 0.05	> 0.05
F-value	0.60	2.68	2.17	1.89	1.00
df1	3	3	3	3	3
df2	66	67	67	64	60
Swing time variability					
p-value	> 0.05	> 0.05	> 0.05	> 0.05	> 0.05
F-value	0.50	2.01	2.22	1.76	1.17
df1	3	3	3	3	3
df2	24	65	67	34	36

70 to 80 years old participants

	SP	UP	FP	AW	CW
Gait speed					
p-value	> 0.05	**< 0.001**	**0.001**	**0.04**	**0.006**
F-value	2.53	7.18	5.44	2.78	4.24
df1	3	3	3	3	3
df2	220	221	219	214	206
Normalized gait speed					
p-value	> 0.05	**0.001**	**0.008**	> 0.05	**0.02**
F-value	2.44	5.35	4.04	2.32	3.46
df1	3	3	3	3	3
df2	217	217	216	211	203
Steps/Meter					
p-value	**0.005**	**0.001**	> 0.05	**0.004**	**0.003**
F-value	4.93	5.61	2.12	4.79	5.11
df1	3	3	3	3	3
df2	47	179	206	78	79
Normalized steps/Meter					
p-value	**0.05**	**0.003**	**0.04**	**< 0.001**	**< 0.001**
F-value	2.57	4.75	2.77	6.76	7.75
df1	3	3	3	3	3
df2	217	217	216	211	203

Swing time variability					
p-value	> 0.05	> 0.05	> 0.05	> 0.05	**0.02**
F-value	1.73	1.57	2.30	1.69	3.47
df1	3	3	3	3	3
df2	47	180	206	78	79

Over 80 years old participants

	SP	UP	FP	AW	CW
Gait speed					
p-value	> 0.05	> 0.05	**0.02**	**0.002**	> 0.05
F-value	1.19	2.33	3.27	5.24	2.28
df1	3	3	3	3	3
df2	212	228	219	199	201
Normalized gait speed					
p-value	> 0.05	> 0.05	**0.02**	**0.003**	> 0.05
F-value	1.23	2.23	3.25	4.87	2.47
df1	3	3	3	3	3
df2	210	225	216	196	197
Steps/Meter					
p-value	> 0.05	> 0.05	**0.009**	> 0.05	> 0.05
F-value	0.12	2.30	4.00	0.05	0.58
df1	3	3	3	3	3
df2	25	129	190	32	43
Normalized steps/Meter					
p-value	> 0.05	**0.05**	**0.05**	**0.003**	**0.01**
F-value	1.05	2.67	2.58	4.90	3.80
df1	3	3	3	3	3
df2	207	222	214	196	196
Swing time variability					
p-value	> 0.05	> 0.05	> 0.05	> 0.05	> 0.05
F-value	0.29	0.74	1.60	0.73	1.15
df1	3	3	3	3	3
df2	25	129	188	32	43

CDR: Clinical Dementia rating. UP: Usual pace, FP: Fast pace, SP: slow pace, AW: animal reciting walk, CW: counting walk.p-value significant when ≤0.05 (in bold) CDR: Clinical dementia rate
