# Supplementary material for: Gait characteristics under different walking conditions: Association with the presence of cognitive impairment in community-dwelling older people
Source: PLoS One. 2017 Jun 1;12(6):e0178566. doi: 10.1371/journal.pone.0178566 (PMC5453541; doi:10.1371/journal.pone.0178566)
Supplement: S4 Table — (PDF) [file pone.0178566.s004.pdf]

Table 4. Simple logistic regression analysis for Model 1 and 2

Model 1

Test mode	UP	FP	SP	CW	AW
	AUC value	AUC value	AUC value	AUC value	AUC value
Gait speed	0.69	0.68	0.64	0.66	0.66
Normalised gait speed	0.69	0.68	0.63	0.66	0.66
Normalised steps per meter	0.69	0.67	0.65	0.67	0.68
Steps per meter	0.69	0.65	0.67	0.69	0.68
Swing time variability	0.64	0.64	0.63	0.67	0.65

Model 2

Test mode	UP	FP	SP	CW	AW
	AUC value	AUC value	AUC value	AUC value	AUC value
Gait speed	0.72	0.70	0.68	0.71	0.69
Normalised gait speed	0.70	0.70	0.67	0.69	0.68
Normalised steps per meter	0.71	0.70	0.69	0.71	0.73
Steps per meter	0.71	0.69	0.60	0.66	0.63
Swing time variability	0.66	0.66	0.64	0.67	0.65
Cycle time variability	0.66	0.66	0.70	0.65	0.63

Walk modes: UP usual pace, FP fast pace, SP slow pace, CW count walk, AW animal walk, AUC: area under the curve AUC:Area under the Curve
