# Supplementary material for: Gait characteristics under different walking conditions: Association with the presence of cognitive impairment in community-dwelling older people
Source: PLoS One. 2017 Jun 1;12(6):e0178566. doi: 10.1371/journal.pone.0178566 (PMC5453541; doi:10.1371/journal.pone.0178566)
Supplement: S5 Table — (PDF) [file pone.0178566.s005.pdf]

		Total study population					70 to 80 years old participants					>80 years old participants				
		CHI	Dementia stages				CHI	Dementia stages				CHI	Dementia stages			
CDR group			MCI	Mild	Moderate	p-value		MCI	Mild	Moderatie	p-value		MCI	Mild	Moderate	p-value
	n	77	140	222	96		37	60	71	29		22	62	130	63	
Variables																
	Units															
Gait speed UW	m/sec	1.05±0.26	0.95±0.23	0.84 ± 0.25	0.78 ± 0.23	**<0.001**	1.06±0.22	0.99±0.22	0.89 ± 0.21	0.88 ± 0.23	**0.03**	0.95 ± 0.25	0.85±0.24	0.76 ± 0.25	0.73 ± 0.22	**0.001**
Number <0.8 m/s		16 (21%)	38(27%)				4 (10.5%)	12(20%)				8(36%)	27(43.5%)			
Gait speed FW	m/sec	1.47±0.38	1.34±0.37	1.16 ± 0.36	1.13 ± 0.27	**<0.001**	1.52±0.34	1.36±0.39	1.29 ± 0.30	1.23 ± 0.27	**0.004**	1.32 ± 0.30	1.22±0.34	1.06 ± 0.33	1.09 ± 0.27	**<0.001**
Gait speed SW	m/sec	0.62 ± 0.16	0.56±0.17	0.55 ± 0.17	0.54 ± 0.18	0.11	0.66±0.16	0.54±0.18	0.56 ± 0.15	0.58 ± 0.19	0.86	0.55 ± 0.14	0.55±0.16	0.50 ± 0.16	0.49 ± 0.16	0.05
Gait speed CW	m/sec	0.78 ± 0.29	0.69±0.26	0.57 ± 0.23	0.51 ± 0.21	**<0.001**	0.76 ± 0.29	0.69±0.27	0.61 ± 0.21	0.53 ± 0.20	**<0.001**	0.57 ± 0.24	0.60±0.25	0.55 ± 0.23	0.49 ± 0.21	0.04
Gait speed AW	m/sec	0.71 ± 0.31	0.64±0.27	0.56 ± 0.25	0.48 ± 0.21	**<0.001**	0.70 ± 0.32	0.65±0.27	0.65 ± 0.23	0.57 ± 0.22	0.18	0.68 ± 0.20	0.58±0.24	0.51 ± 0.23	0.39 ± 0.18	**<0.001**
Nor Gait speed UW	m/sec	1.16 ± 0.28	1.07±0.25	0.94 ± 0.28	0.88 ± 0.27	**<0.001**	1.15 ± 0.24	1.12±0.23	0.98 ± 0.25	0.97 ± 0.26	**<0.001**	0.95 ± 0.25	0.97±0.28	0.89 ± 0.27	0.85 ± 0.24	**0.01**
Nor Gait speed FW	m/sec	1.60 ± 0.40	1.47±0.39	1.31 ± 0.39	1.30 ± 0.31	**<0.001**	1.61 ± 0.36	1.53±0.39	1.45 ± 0.34	1.39 ± 0.29	**0.01**	1.43 ± 0.30	1.36±0.37	1.22 ± 0.37	1.25 ± 0.31	**0.01**
Nor Gait speed SW	m/sec	0.69± 0.18	0.62±0.19	0.61 ± 0.18	0.61 ± 0.20	0.06	0.70 ± 0.17	0.60±0.19	0.64 ± 0.17	0.67 ± 0.21	0.71	0.63 ± 0.18	0.60±0.18	0.57 ± 0.18	0.58 ± 0.17	0.27
Nor Gait speed CW	m/sec	0.87 ± 0.30	0.74±0.27	0.66 ± 0.25	0.59 ± 0.23	**<0.001**	0.87 ± 0.32	0.77±0.29	0.69 ± 0.24	0.63 ± 0.22	**0.004**	0.67 ± 0.23	0.67±0.26	0.62 ± 0.25	0.57 ± 0.23	0.05
Nor Gait speed AW	m/sec	0.80 ± 0.34	0.71±0.29	0.63 ± 0.27	0.57 ± 0.24	**<0.001**	0.77 ± 0.34	0.72±0.30	0.73 ± 0.27	0.63 ± 0.25	0.22	0.70 ± 0.22	0.66±0.27	0.56 ± 0.25	0.46 ± 0.20	**<0.001**
StepM UW	steps/m	1.7± 0.4	1.9±0.5	2.0 ± 0.7	2.2 ± 1.0	**<0.001**	1.8 ± 0.3	1.8±0.5	1.9 ± 0.4	2.0 ± 0.5	0.20	1.8 ± 0.5	2.0±0.5	2.2 ± 0.7	2.3 ± 1.1	**0.02**
StepM FW	steps/m	1.5 ± 0.4	1.6±0.4	1.8 ± 0.5	1.8 ± 0.5	**<0.001**	1.5 ± 0.3	1.6±0.4	1.7 ± 0.4	1.7 ± 0.5	0.05	1.6 ± 0.4	1.7±0.4	1.9 ± 0.6	1.9 ± 0.6	**0.02**
StepM SW	steps/m	2.1 ± 0.5	2.2±0.6	2.5 ± 0.7	2.5 ± 1.5	**<0.001**	2.1 ± 0.5	2.2±0.6	2.5 ± 0.5	2.2 ± 0.6	**0.01**	2.3 ± 0.6	2.4 ±0.6	2.6 ± 0.7	2.7 ± 1.7	0.21
StepM CW	steps/m	1.9 ± 0.5	2.0±0.6	2.3 ± 0.9	2.5 ± 0.8	**<0.001**	1.9 ± 0.4	1.9±0.6	2.2 ± 0.4	2.4 ± 0.7	**<0.001**	2.1 ± 0.6	2.1±0.6	2.5 ± 1.0	2.6 ± 0.8	**0.002**
StepM AW	steps/m	1.9 ± 0.5	2.1±0.6	2.4 ± 0.9	2.6 ± 0.9	**<0.001**	1.9 ± 0.4	2.1±0.7	2.2 ± 0.5	2.3 ± 0.8	**0.01**	2.0 ± 0.6	2.3±0.6	2.6 ± 1.0	2.9 ± 1.0	**<0.001**
Nor StepM UW	steps/m	1.6 ± 0.3	1.7±0.4	1.8 ± 0.5	1.9 ± 0.9	**<0.001**	1.6 ± 0.3	1.6±0.4	1.7 ± 0.4	1.7 ± 0.5	0.22	1.7 ± 0.4	1.8±0.4	1.9 ± 0.6	2.1 ± 1.0	**0.03**
Nor StepM FW	steps/m	1.3 ± 0.3	1.4±0.3	1.5 ± 0.9	1.6 ± 0.4	**0.03**	1.4 ± 0.3	1.4±0.3	1.5 ± 0.3	1.4 ± 0.4	0.29	1.4 ± 0.4	1.5±0.3	1.7 ± 1.1	1.7 ± 0.5	0.23
Nor StepM SW	steps/m	1.9 ± 0.5	2.0±0.5	2.2 ± 0.6	2.1 ± 1.2	**0.01**	1.9 ± 0.4	2.0±0.5	2.2 ± 0.4	2.0 ± 0.5	**0.01**	2.0 ± 0.6	2.1±0.6	2.3 ± 0.6	2.4 ± 1.4	0.05
Nor StepM CW	steps/m	1.7 ± 0.4	1.8±0.5	2.0 ± 0.7	2.2 ± 0.7	**<0.001**	1.7 ± 0.4	1.7±0.5	1.9 ± 0.4	2.1 ± 0.7	**0.001**	1.8 ± 0.5	1.9±0.5	2.1 ± 0.8	2.4 ± 0.7	**<0.001**
Nor StepM AW	steps/m	1.7 ± 0.5	1.9±0.5	2.1± 0.7	2.2 ± 0.8	**<0.001**	1.7 ± 0.3	1.8±0.5	2.0 ± 0.4	2.1 ± 0.6	**0.001**	1.8 ± 0.6	1.9±0.5	2.3 ± 0.8	2.5 ± 0.9	**<0.001**

Table 5-1. Gait speed and Step per meter and their normalized values in five test modes and in four different dementia stages. Univariate one-way ANOVA. CHI: cognitively healthy individuals = CDR 0; MCI : mild cognitive impairment = CDR 0.5; Mild Dementia = CRD 1, Moderate dementia = CDR >1

		Total study population					70 to 80 years old participants					>80 years old participants				
		CHI	Dementia stages				CHI	Dementia stages				CHI	Dementia stages			
CDR group			MCI	Mild	Moderate	p-value		MCI	Mild	Moderate	p-value		MCI	Mild	Moderate	p-value
	n	77	140	222	96		37	60	71	29		22	62	130	63	
Variables																
	Units															
Step width UW	cm	7.9±3.1	8.5±3.2	8.9 ± 3.8	8.9 ± 3.4	0.14	7.4 ± 2.3	8.4±3.4	8.3 ± 4.1	8.2 ± 2.9	0.66	9.0 ± 3.5	9.8±2.9	9.7 ± 3.7	9.2 ± 3.6	0.61
Step width FW	cm	7.7±3.4	7.9±3.2	8.3 ±3.7	8.0 ± 3.2	0.31	7.0 ± 2.5	7.8±3.3	7.3 ± 4.0	7.0 ± 2.9	0.79	7.7 ± 3.4	8.4±2.8	8.9 ± 3.6	8.4 ± 3.5	0.39
Step width SW	cm	8.9±3.7	9.5±3.7	9.7 ± 4.0	9.8 ± 3.6	0.60	7.2 ± 3.4	9.4±4.0	8.9 ± 4.0	8.8 ± 2.9	0.98	10.4 ± 3.2	10.5±3.1	10.1 ± 4.2	10.3 ± 3.9	0.76
Step width CW	cm	8.4±4.0	9.2±3.6	9.4 ± 4.8	9.6 ± 3.4	0.29	7.8 ± 3.9	8.9±3.9	8.4 ± 4.2	9.0 ± 2.9	0.70	9.8 ± 3.6	10.1±3.1	9.8 ± 5.2	9.8 ± 3.6	0.99
Step width AW	cm	9.1±3.9	9.5±3.9	10.2 ± 4.2	10.6 ± 4.1	**0.04**	8.0 ± 3.7	8.9±3.8	9.1 ± 4.0	9.1 ± 3.5	0.59	10.3 ± 3.7	10.2±3.4	11.0 ± 4.3	12.0 ± 4.3	**0.03**
Step width var UW	%	3.9±2.8	4.7 ± 2.8	4.4 ± 3.5	4.4 ± 3.7	0.40	4.3±2.8	4.7 ± 2.2	4.5 ± 2.9	4.7 ± 3.8	0.95	4.7±2.6	4.8 ± 3.4	4.4 ± 3.8	3.9 ± 3.5	0.40
Step width var FW	%	3.7±2.4	4.0 ± 2.7	4.1 ± 3.0	4.3 ± 3.3	0.51	3.8±2.7	3.9 ± 2.5	4.0 ± 2.4	4.9 ± 3.0	0.12	4.2±2.3	4.0 ± 3.1	4.2 ± 3.2	4.1 ± 3.4	0.90
Step width var SW	%	3.7±2.9	4.8 ± 3.4	4.6 ± 3.4	4.9 ± 3.7	0.73	4.3±3.0	4.7 ± 2.8	4.2 ± 3.3	5.8 ± 3.2	0.09	4.6±3.1	5.1± 4.1	4.5 ± 3.6	4.4 ± 4.1	0.55
Step width var CW	%	3.5±3.1	4.5 ± 3.8	4.7 ± 3.7	5.2 ± 3.3	**0.02**	4.1±3.1	4.1 ± 2.9	4.4 ± 3.5	6.5 ± 3.2	**0.003**	4.6±2.6	5.2 ± 4.7	4.7 ± 3.8	4.4 ± 3.5	0.65
Step width var AW	%	3.7±2.9	4.6 ± 3.4	4.6 ± 4.0	4.8 ± 4.1	0.22	4.7±3.0	4.6 ± 2.9	4.5 ± 3.7	5.4 ± 3.6	0.45	4.7±3.0	4.7 ± 4.0	4.9 ± 4.0	3.9 ± 4.7	0.28
CyTvar UW	%	0.41±0.04	0.40±0.04	0.41 ± 0.05	0.40 ± 0.10	0.32	0.40 ± 0.04	0.41±0.03	0.41 ± 0.04	0.41 ± 0.05	0.24	0.42 ± 0.05	0.40±0.04	0.40 ± 0.05	0.40 ± 0.12	1.00
CyTvar FW	%	0.37±0.03	0.36±0.03	0.36 ± 0.05	0.37 ± 0.42	0.89	0.36 ± 0.03	0.36±0.04	0.36± 0.04	0.38 ± 0.18	0.44	0.38 ± 0.03	0.36±0.03	0.36 ± 0.05	0.36 ± 0.52	NaN
CyTvar SW	%	0.51±0.20	0.48±0.09	0.47 ± 0.07	0.45 ± 0.07	**0.005**	0.49 ± 0.25	0.48±0.10	0. 47 ± 0.08	0. 49 ± 0.07	0.69	0.51 ± 0.09	0.47±0.08	0.46 ± 0.07	0.45 ± 0.06	0.24
CyTvar CW	%	0.48±0.13	0.49±0.85	0.47 ± 0.53	0.46 ± 0.36	0.89	0.48 ± 0.16	0.51±0.54	0.49 ± 0.37	0.47 ± 0.48	0.94	0.48 ± 0.09	0.48±1.17	0.47 ± 0.63	0.46 ± 0.43	0.99
CyTvar AW	%	0.48±0.28	0.48±1.42	0.47 ± 0.70	0.46 ± 0.78	0.98	0.48 ± 0.34	0.48 ±2.1	0.48 ± 0.58	0.47 ± 0.12	1.00	0.49 ± 0.06	0.46±0.65	0.47 ± 0.82	0.45 ± 0.98	0.97
SwTvar UW	%	13.8±2.7	14.8±3.0	15.6 ± 3.7	16.4 ± 3.6	**<0.001**	13.6 ± 2.6	14.5±2.6	15.2 ± 3.0	15.1 ± 3.2	0.31	15.1 ± 2.8	16.2±3.4	16.5 ± 4.0	16.8 ± 3.7	0.23
SwTvar FW	%	11.7±3.0	12.1±2.9	13.0 ± 3.3	13.2 ± 5.7	**0.002**	11.9± 3.1	12.0±2.9	12.4 ± 3.1	12.9 ± 5.9	0.39	12.3 ± 2.9	13.7±2.8	13.8 ± 3.3	13.3 ± 6.7	0.72
SwTvar SW	%	17.0±3.2	18.3±3.2	18.4 ± 3.7	18.4 ± 7.5	0.31	17.0 ± 3.2	18.3±3.2	18.4 ± 3.2	17.9 ± 3.9	0.34	17.5 ± 2.8	19.1±3.2	18.7 ± 3.9	19.6 ± 8.9	0.52
SwTvar CW	%	15.2±4.4	16.6±5.3	17.3 ± 8.0	18.9 ± 13.0	**0.03**	15.5 ± 4.1	16.5±6.7	16.6 ± 4.4	18.3 ± 17.1	0.39	16.8 ± 5.1	18.3±4.1	18.3 ± 9.6	20.1 ± 15.3	0.37
SwTvar AW	%	15.4±4.6	17.1±5.4	17.5 ± 5.8	19.3 ± 6.5	**<0.001**	15.6± ± 4.0	16.8±6.3	16.6 ± 3.7	19.3 ± 7.4	0.43	16.3± 5.7	18.0±4.6	18.6 ± 6.3	20.9 ± 7.4	**0.006**

Table 5-2. Gait characteristics in five test modes and in four different dementia stages. Univariate one-way ANOVA . CHI: cognitively healthy individuals = CDR 0; MCI : mild cognitive impairment = CDR 0.5; Mild Dementia = CRD 1, Moderate dementia = CDR >1

CDR group		Total study population					70 to 80 years old participants					>80 years old participants				
		CHI	Dementia stages				CHI	Dementia stages				CHI	Dementia stages			
			MCI	Mild	Moderate	p-value		MCI	Mild	Moderate	p-value		MCI	Mild	Moderate	p-value
	n	77	140	222	96		37	60	71	29		22	62	130	63	
Variables	Units															
DTC Nor speed CW	%	22.7 ± 21.3	24.3±21.2	25.0 ± 21.7	30.9 ± 20.2	0.05	23.1±23.0	23.8±23.1	24.7 ± 21.4	32.5 ± 18.8	0.138	19.5 ± 17.0	24.3±18.7	24.1 ± 21.9	30.3 ± 21.2	0.068
DTC Step width CW	%	-5.1 ± 30.6	-4.6±48.5	-5.7 ±67.5	-11.3 ± 28.8	0.588	-3.8±35.1	-2.5±59.5	-2.2 ± 42.5	-12.7 ± 23.8	0.517	-11.0 ± 26.0	-9.8±39.6	-5.7 ± 81.72	-13.5 ± 30.0	0.696
DTC Step width var CW	%	2.1 ± 201.7	0±181.9	-2.2 ± 180.5	-6.2 ± 336.0	0.974	2.5 ± 45.1	5.7±64.3	1.3 ± 66.0	-9.1 ± 127.6	0.729	2.1 ± 62.5	-4.3±267.8	-3.0 ± 229.6	-3.3 ± 397.8	1
DTC CyTvar CW	%	-16.7 ±30.0	-21.2±76.9	-14.9 ± 135.6	-14.5 ± 100.9	0.897	-18.0 ± 36.3	24.4±113.5	-16.7± 108.0	-14.6 ± 136.5	0.943	-13.8 ± 20.4	-17.5±17.3	-11.8 ± 157.7	-14.7 ± 120.6	0.959
DTC SwTvar CW	%	-6.8 ± 17.1	-9.1±32.2	-11.3 ± 47.1	-15.5 ± 28.2	0.43	-9.5 ± 19.0	-8.6±45.4	-10.4 ± 23.6	-15.6 ± 13.1	0.530	-8.2 ± 18.3	-9.1±16.5	-12.1 ± 58.6	-14.8 ± 33.3	0.738
DTC StepM CW	%	-6.3 ± 13.8	-7.1±17.1	-8.3 ± 19.6	-15.1 ± 34.7	**0.02**	-7.2 ± 16.5	-7.1±21.3	-8.2 ± 14.6	-9.7 ± 24.1	0.768	-10.2 ± 11.5	-5.3±13.1	-10.8 ± 22.4	-14.8 ± 37.4	0.170
DTC N StepM CW	%	-7.4 ± 14.0	-7.1±17.4	-7.9 ± 19.6	-15.3 ± 34.0	**0.007**	-7.6 ± 16.5	-7.1±21.6	-8.1 ± 14.6	-9.9 ± 24.9	0.833	-10.5± 11.5	-5.5±13.4	-9.9 ± 22.3	-16.0 ± 36.2	0.099
DTC Nor speed AW	%	24.6 ± 23.3	27.1±22.9	28.6 ± 23.9	36.4 ± 20.3	**0.003**	30.2 ± 26.8	26.4±22.8	24.0 ± 22.5	31.2 ± 19.0	0.278	24.3 ± 13.8	27.0±21.3	31.9 ± 24.4	40.4 ± 20.8	**0.001**
DTC Step width AW	%	-8.6 ± 49.2	-10.1±34.8	-12.5 ± 115.4	-25.0 ± 35.1	0.47	-4.2 ± 63.4	-10.7±33.4	-16.4 ± 44.1	-23.7 ± 27.7	0.267	-13.4 ± 31.8	-11.5±32.5	-12.5 ± 148.2	-30.8 ± 37.8	0.461
DTC Step width var AW	%	0± 121.6	-1.3±135.0	1.0 ± 134.5	-11.8 ± 252.7	0.802	6.3 ± 152.2	1.3±78.7	5.6 ± 81.2	-13.1 ± 174.1	0.715	-12.5 ± 46.8	-6.8±182.1	-13.3 ± 34.1	-9.6 ± 44.2	0.889
DTC CyTvar AW	%	-17.1 ± 54.7	-17.5±31.2	-13.6 ± 34.9	-11.6 ± 36.5	0.59	-25.7 ± 58.8	-19.1±27.4	-12.6 ± 34.5	-13.7 ± 23.5	0.399	-15.6 ± 13.5	-15.0±18.4	-13.3 ± 34.1	-9.6 ± 44.2	0.579
DTC SwTvar AW	%	-9.9 ± 23.6	-11.6±23.6	-13.9 ± 21.7	-19.7 ± 22.6	**0.02**	-10.6 ± 24.1	-10.3±27.1	-10.4 ± 18.4	-19.3 ± 22.8	0.124	--8.6 ± 19.5	-14.4±21.6	-19.0 ± 23.7	-20.0 ± 22.5	0.12
DTC StepM AW	%	-8.1 ± 15.6	-10.5±20.3	-11.4 ± 19.6	-19.6 ± 28.7	**0.001**	-7.5 ± 19.5	-9.5±23.3	-8.9 ± 19.8	-14.9 ± 23.7	0.279	-9.5 ± 9.6	-10.9±15.1	-13.0 ± 19.8	-20.4 ± 31.2	0.11
DTC N StepM AW	%	-7.7 ± 15.3	-10.6±20.1	-12.1 ± 23.3	-19.6 ± 28.6	**0.001**	-7.7 ± 18.9	-8.2±22.7	-9.0 ± 18.8	-15.3 ± 23.9	0.228	-8.7 ± 9.6	-11.0±15.3	-14.1 ± 26.0	-20.4 ± 31.0	0.14

Table 5-3. Dual task cost between dual task walk (CW and AW) and usual walk test ((CW variable –UW variable)/CW variable) for the gait characteristics in four different dementia stages. Univariate one-way ANOVA CHI: cognitively healthy individuals = CDR 0; MCI : mild cognitive impairment = CDR 0.5; Mild Dementia = CRD 1, Moderate dementia = CDR >1.
